# Willingness to pay for social health insurance and its determinants among public servants in Mekelle City, Northern Ethiopia: a mixed methods study

**DOI:** 10.1186/s12962-019-0171-x

**Published:** 2019-01-15

**Authors:** Meles Tekie Gidey, Gebremedhin Beedemariam Gebretekle, Mary-Ellen Hogan, Teferi Gedif Fenta

**Affiliations:** 1 School of Pharmacy, College of Health Sciences, Mekelle University, Mekelle, Ethiopia; 2 School of Pharmacy, College of Health Sciences, Addis Ababa University, Addis Ababa, Ethiopia; 3 Leslie Dan Faculty of Pharmacy, University of Toronto, Toronto, Canada

**Keywords:** Willingness to pay, Health insurance, Contingent valuation method, Universal health coverage, Ethiopia

## Abstract

**Background:**

Owing to lack of adequate healthcare financing, access to at least the basic health services is still a problem in Ethiopia. With the intention of raising funds and ensuring universal health coverage, a mandatory health insurance scheme has been introduced. The Community Based Health Insurance has been implemented in all regions of the country, while implementation of social health insurance was delayed mainly due to resistance from public servants. This study was, therefore, aimed to assess willingness to pay for social health insurance and its determinant factors among public servants in Mekelle city, Northern Ethiopia.

**Methods:**

A concurrent mixed approach of cross-sectional study design using double bound dichotomous choice contingent valuation method and qualitative focus group discussions was employed. A total 384 public servants were recruited from randomly selected institutions and six focus group discussions (n = 36) were carried out with purposively selected respondents. Participants’ mean willingness to pay (WTP) and independent predictors of WTP were identified using an interval data logit model. Qualitative data were analyzed using thematic analysis.

**Results:**

From the 384 participants, 381 completed the interview, making a response rate of 99.2%. Among these respondents 85.3% preferred social health insurance and were willing to pay for the scheme. Their estimated mean WTP was 3.6% of their monthly salary. Lack of money to pay (42.6%) was the major stumbling block to enrolling in the scheme. Respondents’ WTP was significantly positively associated with their level of income but their WTP decreased with increasing age and educational status. On the other hand, a majority of focus group discussion participants were not willing to pay the 3% premium set by the government unless some preconditions were satisfied. The amount of premium contribution, benefit package and poor quality of health service were the major factors affecting their WTP.

**Conclusion:**

The majority of the public servants were willing to be part of the social health insurance scheme, with a mean WTP of 3.6% of their monthly salary. This was greater than the premium proposed by the government (3%). This can pave the way to start the scheme but attention should focus on improving the quality of health services.

**Electronic supplementary material:**

The online version of this article (10.1186/s12962-019-0171-x) contains supplementary material, which is available to authorized users.

## Background

Significant proportions of people all over the world suffer and die due to lack of access to basic healthcare services. In low and middle-income countries alone, 150 million people suffer a health-related financial catastrophe each year, and 100 million people (the equivalent of three people every second) are pushed into poverty as a result of out of pocket (OOP) health expenditures [1–3]. OOP health expenditure is the major source of health care financing in low income countries and those with emerging economies compared to developed nations. Individuals in countries with emerging economies paid 75.1% to 97.7% of their private healthcare expenditures [4]. In addition, the coverage of health services in these countries is constrained by inadequate funding [5, 6]. High reliance on OOP payment and high dependency on funding from development assistance are the main reason for lack of funding for health in low and middle income countries [7].

This indicates that health service fees are a major obstacle to healthcare coverage and utilization [8], and the only way to reduce reliance on direct OOP payments and to attain Universal Health Coverage (UHC) for governments is to encourage the risk-pooling prepayment mechanisms [9, 11]. In 2005, WHO urged member states to “ensure that health-financing systems introduce or develop prepayment of financial contributions for the health sector, with a view to sharing risk among the population and avoiding catastrophic healthcare expenditure and impoverishment of individuals as a result of seeking care” [10]. In connection with this, many low and middle income countries are considering on how to reform their health care systems to provide effective financial risk protection for all, as part of UHC [1, 3]. Introducing Social Health Insurance (SHI) was considered as one of the most powerful risk pooling mechanisms in most developing countries to achieve UHC. The UHC had been achieved in many countries in the world by establishing SHI as the country’s health care financing mechanism [11].

Like many other low income countries, healthcare financing has been a major challenge for Ethiopians. Government expenditure on health as a percentage of total expenditure was 5.4% which is below the targets set by Abuja Declaration of 15% [12] and 90.6% of the total health expenditure is funded from household OOP expenditures [13]. Per capita annual national health expenditure was US$21 in 2010/11 which was far below the WHO recommendation of US$44 per capita for low-income countries [14]. The national health expenditure is expected to reach US$212 in 2040, with government spending 4.6% of GDP, which will be still lower than the expected average spending of lower income countries (6.7%) [8].

As a result of financial constraints for health, the Ethiopian Federal Ministry of Health began health care financing reform in 1998 to improve and diversify resource mobilization for health and secure financial protection for its citizens. Implementing a health insurance scheme was one of nine intervention strategies mentioned in this reform [15]. The government developed a health insurance strategy in 2008, and two types of health insurance have been proposed since 2010, Community Based Health Insurance and Social Health Insurance (SHI) [16, 17].

The Community Based Health Insurance scheme was intended to cover approximately 89% of the population who are mainly rural dwellers. The SHI was intended to cover the employed and their family members, approximately 11% of the population (public servants, permanent employees working in private organizations and pensioners). Enrollment in SHI is compulsory and the proposed contribution is 3% of their salary [17]. The insurance benefit package includes outpatient care, inpatient care, delivery services, surgical service, diagnostic tests and generic drugs included in the drug list of the health insurance agency. Treatment outside Ethiopia, treatment related to drug abuse or addiction, periodic medical checkups unrelated to illness, cosmetic surgery, dentures, implants, crowns, organ transplants, dialysis except acute renal failure, provision of eye glasses, contact lenses and hearing aids are excluded from the benefit packages [18].

Despite the government’s plan to fully implement SHI by 2014 [19], it has been repeatedly postponed, largely due to strong resistance from public servants. Hence, this study was conducted to explore public servants’ WTP and factors contributing to resistance to SHI. The study focused on health professionals, teachers and support staff employed in health facilities and schools. Understanding the views of teachers and health professionals is important because of their influence in the society which could be either positive or negative. Support staffs were also enrolled in the study to gain information about the perspectives of the relatively lower income segments of the public servants on SHI.

## Methods

The study employed a mixed methods approach using a contingent valuation study and a qualitative study using focus group discussions (FGDs). The study was conducted in Mekelle City, Tigray Regional State, Northern Ethiopia between April and May, 2017. Respondents were recruited from schools and health facilities. Employees must have worked for at least 6 months and be willing to participate in the study.

A sample size of 384 was calculated for the cross-sectional survey using a single population proportion formula [19]; assuming 50% of the public servants are willing to pay with 95% CI and 5% margin of error. A proportional number of respondents were drawn from each institution (four high schools, eight elementary schools, two hospitals, and five health centers) which were selected using systematic random sample technique. An interviewer-administered structured questionnaire was adapted from other studies [19–21] and pretested among 29 public servants prior to the actual data collection and modification was done accordingly (Additional file 1). The questionnaire included participants’ socio-demographics, healthcare utilization and hypothetical healthcare financing scenarios to determine their WTP. The English version questionnaire was translated into local language (Tigrigna) and back translated into English for consistency. Data were collected by three trained pharmacists.

Following the quantitative survey, six FGDs (6 discussants each, n = 36) were carried out to obtain information regarding participants’ perceived affordability of healthcare costs, knowledge and understanding of health insurance, WTP for SHI and concerns about SHI implementation. The FGD participants were comprised of two FGDs with teachers, two with health professionals and two with support staff. The head of each institution was consulted in choosing the FGD participants. The discussants did not participate in the quantitative survey. The first author (MTG) and trained research assistant facilitated all FGDs using a semi-structured interview guide with flexible probing techniques (Additional files 2 and 3). All discussions were tape recorded and transcribed verbatim. Each FGD lasted 45 to 80 min with a mean of 55 min.

### Choice of WTP method

The double bound dichotomous choice (DBDC) approach was used to estimate the WTP as it has good statistical efficiency and is simple to conduct, and it has been extensively used in the valuation of non-marketed goods [22]. In contingent valuation, first the hypothetical market is described to respondents and a series of questions was asked. Three hypothetical scenario choice sets were adapted from previous studies in Ethiopia [20, 23]. The set of choices were scenario A, which was no insurance; scenario B- compulsory insurance (social health insurance); scenario C -voluntary insurance (Additional file 1). In this method, the respondent only answers ‘yes’ or ‘no’ to a given question about the WTP amount [24]. Different starting bids identified from the pretest (i.e. 2, 3, 4 and 6%) were distributed randomly to participants and the respondent was asked whether he/she was willing to pay for a specified bid amount. If the respondent says ‘yes’ to the first bid, a second bid that was twice as much would be offered. If the respondent says “No” to the first bid, a second lower bid (1/2 first bid) would be offered. The first bid amount was distributed to the respondents randomly to minimize starting point bias.

Assuming a linear functional form for the WTP, the econometric model is:

1 $${\text{WTP}}_{\text{i}} \left( {{\text{z}}_{\text{i}} ,{\text{ u}}_{\text{i}} } \right)\, = \,{\text{ z}}_{\text{i}} \upbeta \, + \,{\text{u}}_{\text{i}}$$

where z_i_ is a vector of explanatory variables, β is a vector of parameters and u_i_ is an error term assumed to be independently and randomly distributed with mean zero and constant variance, σ^2^.

Let the first bid amount be t_1_ and the second one t_2_, and then each individual will be in one of the following categories:

1. The individual answers ‘yes’ to the first question and ‘no’ to the second, then t_2_ > t_1_. In this case we can infer that t_1_ ≤ WTP < t_2_.
2. The individual answers yes to the first question and yes to the second, then t_2_ ≤ WTP < $\infty$.
3. The individual answers no to the first question and yes to the second, then t_2_ < t_1_. In this case we have t_2_ ≤ WTP < t_1_.
4. The individual answers no to the first and second questions, then we have 0 < WTP < t_2_.

Then, the probability of each of the four cases is defined as:

A. 2 $$Pr\left( {t_{1} \le WTP < t_{2} } \right) = {\text{Pr }}\left( {\frac{{t_{1} - z_{i}^{'} \beta }}{\sigma } \le \frac{{u_{i} }}{\sigma } < \frac{{t_{2} - z_{i}^{'} \beta }}{\sigma }} \right) = \Phi \left( {z_{i }^{'} \frac{\beta }{\sigma } - \frac{{t_{1} }}{\sigma }} \right)\, - \,\Phi \left( {z_{i }^{'} \frac{\beta }{\sigma } - \frac{{t_{2} }}{\sigma }} \right)$$
B. 3 $$\Pr \left( {WTP > t_{1} , \,WTP > t_{2} } \right) = \Phi \left( {z_{i }^{\prime} \frac{\beta }{\sigma } - \frac{{t_{2} }}{\sigma }} \right)$$
C. 4 $$\Pr \left( {t_{2} \le WTP < t_{1} } \right) = \Phi \left( {z_{i }^{\prime} \frac{\beta }{\sigma } - \frac{{t_{2} }}{\sigma }} \right) - \Phi \left( {z_{i }^{'} \frac{\beta }{\sigma } - \frac{{t_{1} }}{\sigma }} \right)$$
D. 5 $$Pr\left( {WTP < t_{1} , WTP < t_{2} } \right) = 1 - \Phi \left( {z_{i }^{\prime} \frac{\beta }{\sigma } - \frac{{t_{2} }}{\sigma }} \right)$$

Estimation of β and σ was based on the maximum likelihood method. The function that needs to be maximized to find the parameters of the model is:

6 $$\sum\limits_{i = 1}^{N} {\left[ {d_{i}^{yn} ln\left( {\Phi \left( {z_{i }^{'} \frac{\beta }{\sigma } - \frac{{t_{1} }}{\sigma }} \right) - \Phi \left( {z_{i }^{'} \frac{\beta }{\sigma } - \frac{{t_{2} }}{\sigma }} \right)} \right) + d_{i}^{yy} ln\left( {\Phi \left( {z_{i }^{'} \frac{\beta }{\sigma } - \frac{{t_{2} }}{\sigma }} \right)} \right) + d_{i}^{ny} ln\left( {\Phi \left( {z_{i }^{'} \frac{\beta }{\sigma } - \frac{{t_{2} }}{\sigma }} \right) - \Phi \left( {z_{i }^{'} \frac{\beta }{\sigma } - \frac{{t_{1} }}{\sigma }} \right)} \right) + d_{i}^{nn} ln\left( {1 - \Phi \left( {z_{i }^{'} \frac{\beta }{\sigma } - \frac{{t_{2} }}{\sigma }} \right)} \right)} \right]}$$

where $d_{i}^{yn} , \,d_{i}^{yy}$, $d_{i}^{ny} , \,d_{i}^{nn}$ are indicator variables that take the value of one or zero depending on the relevant case for each individual. Each respondent contributes to the logarithm of the likelihood function in only one of its four parts. Hence, we obtain directly β and σ then we can estimate WTP [25].

### Data analysis

The quantitative data were analyzed using Stata version 12.0. Participants’ socio-demographic characteristics, household members’ chronic disease status, their occupation, presence of free health coverage and prior information on health insurance were the factors assessed for predicting WTP. Their mean WTP and predictors of WTP were identified using the interval data logit model using the ‘doubleb’ command in Stata 12 as explained by Lopez–Feldman [25]. The significance level was set at 95% confidence interval and *p*-value < .05. The qualitative data was manually analyzed employing thematic analysis. Authors MTG and GBG in collaboration with authors TGF and MEH carried out the analysis and interpretation of the data. Finally, findings were shared with six of the participants (one each from the FGDs) and they confirmed that the interpretations were reflective of their insight and experiences (Additional file 4).

## Results

### Socio-demographic characteristics of the study participants

From the 384 participants, 381 participated in the interview, making a response rate of 99.2%. The majority (60.1%) of participants was females and their mean (± SD) age was 37 (± 9.2) years. The average family size and monthly household income of participants was 3.4 (± 1.8) and 5423 (± 3165) Ethiopia Birr (ETB) (1US$ = 23ETB), respectively. A summary of socio-demographic characteristics is presented in Table 1.

**Table 1:** Socio-demographic characteristics of public servants in Mekelle City, Northern Ethiopia, 2017

Variables	Survey participants		Focus group participants	
	Frequency	Percentage	Frequency	Percent
*Gender*				
Male	152	39.9	21	58.3
Female	229	60.1	15	41.7
*Age category*				
< 30	91	23.9	11	30.6
30–39	146	38.3	12	33.3
> 40	144	37.8	13	36.1
*Marital status*				
Not married	147	38.6	13	36.1
Married	234	61.4	23	63.9
*Respondents educational status*				
Elementary school	23	6.0		
Diploma certificate	153	42.5	19	52.8
Degree and above	196	51.5	17	47.2
*Occupation*				
Teacher	171	44.9	12	33.3
Health professional	125	32.8	12	33.3
Supportive staff	85	22.3	12	33.3
*Household family size*				
≤ 3	186	48.8	17	47.2
4–6	178	46.7	14	38.9
≥ 7	17	4.5	5	13.9
*Homes with children under age 5* *years*				
No	257	67.5	21	58.3
Yes	124	32.5	15	41.7

### Health and health care related characteristics of the study participants

One hundred and sixty (42.0%) respondents had at least one episode of acute illness in the last 12 months and almost all (98.1%) of them sought treatment for their recent episodes. Of the total participants, 282 (74.0%) of respondents’ healthcare expenditure was OOP and 306 (80.3%) of them reported that it was unaffordable. At the same time, about half (51.7%) of respondents were not satisfied with the quality of health services being rendered in public health facilities (Table 2).

**Table 2:** Health and health related situations among public servants in Mekelle City, Northern Ethiopia, 2017

Description	Frequency	Percent
*Presence of chronic illness among family members (n = 381)*		
Yes	71	18.6
*Presence of acute illness in a family member in 12* *months period (n = 302)*^a^		
Yes	136	45.0
*Presence of any acute illness on the respondent within 12* *months period (n = 381)*		
Yes	160	42.0
*Seeking treatment for the recent episode of illness (n = 160)*		
Yes	157	98.1
*Place of treatment sought (n = 157)*		
Private health facility	25	15.9
Public health facility	132	84.1
*Healthcare expenditure (n = 381)*		
Out of pocket	282	74.0
Employer	67	17.5
Others (free, civic society)	33	8.5
*Affordability of health care costs (n = 381)*		
Affordable	75	19.7
Not affordable	306	80.3
*Satisfaction with quality of services at public health facilities (n = 381)*		
Dissatisfied	197	51.7
Neutral	37	9.8
Satisfied	147	38.6
*Satisfaction with the cost of services at public health facilities (n = 381)*		
Dissatisfied	120	31.5
Neutral	27	7.1
Satisfied	234	61.4

^a^Questions were not answered by all participants

### Respondents’ preference for health care financing options

The majority 336 (88.2%) of respondents agreed on the need to introduce SHI. Regarding their preference of health care financing, 325 (85.3%) of them preferred the mandatory SHI package; while 11 (2.9%) of them preferred voluntary health insurance. In general, 336 (88.2%) of the respondents were supportive of introducing a health insurance scheme, either voluntary or mandatory. However, 45 (11.8%) participants preferred OOP health expenditure (Additional file 4).

### Respondents’ willingness to pay for social health insurance

**Fig. 1:**
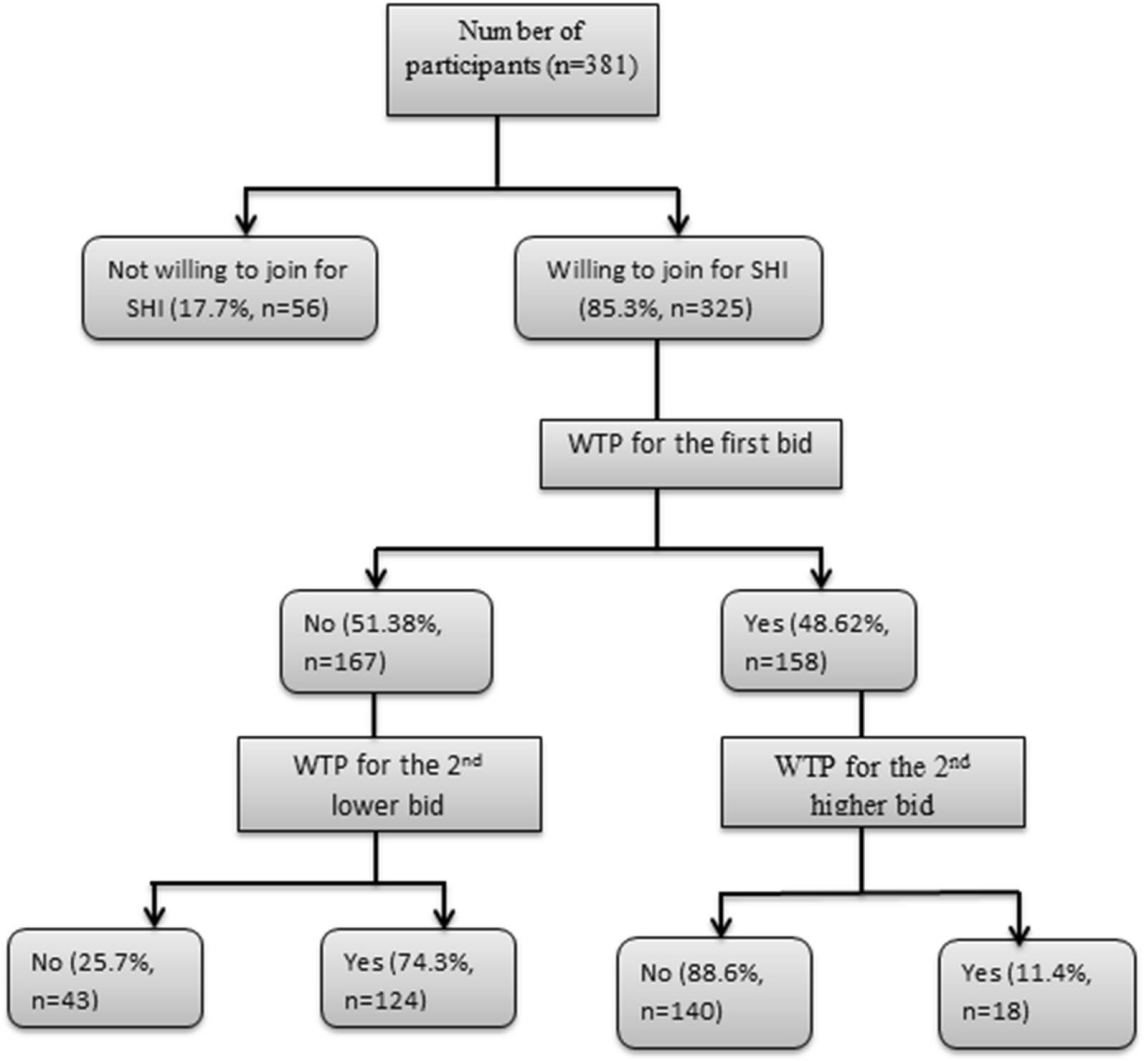
Summary statistics to double-bounded dichotomous choice questions

Regarding the initial bid distribution, 77 (23.69%), 82 (25.2%), 80 (24.6%) and 86 (26.4%) of the participants picked 2, 3, 4 and 6% initial bid amounts, respectively. About 48.6% of them responded “yes” to the first bid (Fig. 1).

**Fig. 2:**
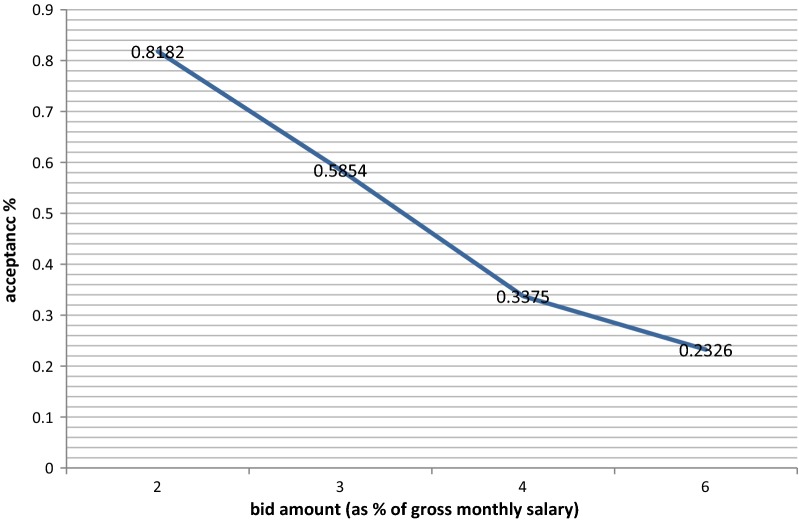
Respondents bid acceptance rate (%) and bids amounts (as % of gross monthly salary) (using double bounded dichotomous choice method)

Across the contingent valuation question, the amount of first bid was an important factor behind participants WTP. As the bid amount increased, the probability of acceptance decreased (Fig. 2).

The large majority (85.3%) of public servants were willing to join and pay for SHI. The overall estimated mean WTP was 3.6% (95% CI 3.4–3.8) of their gross monthly salary. Access to free health services 277 (85.2%), financial security in times of ill health 278 (85.5%) and helping others who can’t afford their medical bills 238 (73.2%) were mentioned as the key drivers for their WTP. For those who were not willing to join 56 (14.7%), lack of money to pay was raised as the main reason 23 (41.1%) (Table 3).

**Table 3:** Reasons behind public servants’ willingness/unwillingness to pay for social health insurance in Mekelle City, Northern Ethiopia, 2017

Variable	Frequency	Percent
*Reasons for joining SHI (n = 325)* ^a^		
To get free health service at point of service provision	277	85.2
To help others who can’t afford their medical costs	238	73.2
For security and peace of mind in times of ill-health	278	85.5
Facing health problem frequently	14	4.3
*Reasons for not joining SHI (n = 56)* ^a^		
Lack of money to pay	23	41.1
It doesn’t cover all health services	12	21.4
OOP charge is better	12	21.4
Lack of trust in insurance scheme	17	30.4
Poor quality of health service in public health facility	9	16.1
Others (double payment from wife and husband, government should pay, I have free card)	5	8.9

^a^Multiple answers possible (total may exceed 100%)

### Predictors of willingness to pay for social health insurance

Respondents’ WTP was significantly associated with their age, educational status and household income. In this regard, older age and more educated respondents were willing to pay less as compared to their younger counterparts. But, as respondents’ household income increased, the amount they were willing to pay also increased. The result of the model is presented in Table 4.

**Table 4:** The effect of explanatory variables on public servants’ willingness to pay for social health insurance in Mekelle City, Northern Ethiopia, 2017

Number of obs = 325	
Wald χ^2^ (11) = 20.23	
Prob > χ^2^ = .0423	
Log likelihood = − 366.11174	

	Coef.	Std. err	Z	P > |z|	(95% conf. interval)	
Age	− .045709	.0152124	− 3.00	.003	− .0755247	− .0158933
Gender	− .4345219	.2435418	− 1.78	.074	− .9118551	.0428112
Profession	− .0581826	.2500453	− .23	.816	− .5482623	.431897
Marital status	− .3926512	.3244671	− 1.21	.226	− 1.028595	.2432927
Having a child under 5 years	. 4000567	.28163	1.42	.155	− .151928	.9520413
Education	− .5756118	.2441041	− 2.36	.018	− 1.054047	− .0971765
Free health coverage	− .0418736	.2641692	− .16	.874	− .5596358	.4758886
Insurance awareness	− .3827888	.3542665	− 1.08	.280	− 1.077138	.311560
Chronic disease	− .1424357	.2984176	− .48	.633	− .7273234	.4424521
Family size	.1399768	.0992248	1.41	.158	− .0545002	.3344538
Household income	.0001064	.0000428	2.48	.013	.0000225	.0001904
_cons	5.148927	.5623156	9.16	.000	4.046809	6.251045
Sigma_cons	1.70698	.0892299	19.13	.000	1.532093	1.881868

### Qualitative findings

A total of six FGDs were conducted and three major themes were emerged from the thematic analysis: affordability of health services, participants’ knowledge of health insurance and factors influencing WTP for SHI.

### Affordability of health services

For the majority of the participants, OOP payment was a means to cover their health expenditure but very few of them had employment-based healthcare coverage. All participants agreed that healthcare was expensive and as a result, significant numbers of participants failed to seek medical care on time, due to a shortage of money. They reported that they cannot afford their medical bills unless they borrow from relatives. This has been illustrated by one teacher:

*“Healthcare costs are very expensive, unaffordable and I don’t think they consider the income of most employees. Considering my income and affordability of health services, having a serious health problem is similar to death sentence (Male, 59* *years, Teacher)”.*

On the other hand, some of the participants indicated that the cost of health services was fair in public health facilities compared to private ones, but the lack of some diagnostic tests, poor service quality and shortages of medications in the public system were major aggravating factors for extra expense and lack of access as compared to private hospitals. One support staff from a health institution stated that:

*“The cost of medication is lower in public hospitals but there is a frequent shortage of vital medicines. When the private pharmacies know that a particular medicine is out of stock from public health facilities, they would immediately increase the price way too high. This forced us to pay an extra high cost, which is unaffordable to many public servants (Female, 27* *years, Support staff).”*

### Knowledge of health insurance

The majority of participants had good knowledge regarding what health insurance is, how it works and its concepts and purpose. They viewed health insurance as crucial to access to healthcare for all citizens regardless of their socioeconomic status. Most participants believed that everyone can benefit from SHI but a few of them strongly argued that only the poor are beneficiaries of the scheme. This was illustrated by one participant:

*“Health insurance is about helping each other in times of ill health based on prior contributions. Because none of us is certain about our health status, it is important to have a guarantee for everyone: all people whether rich or poor may not have money at hand in times of emergency health conditions. It is amazing that every car in Ethiopia has insurance but we don’t have health insurance for our precious life (Male, 42* *years, Health professional)”.*

### Factors influencing willingness to pay for social health insurance

Once health expenditure and knowledge of health insurance was explored; the principle, purpose and benefit packages of SHI were explained. Perceived need and factors affecting participants’ WTP were then discussed. Four sub-themes emerged: premium contribution; benefit package, quality of health services and eligibility of family members to be covered.

### Premium contribution

We used a 3% premium contribution, the premium set by the Ethiopian Health Insurance Agency, to elicit participants’ WTP. Despite their support to implement of SHI, a majority of the participants were not willing to contribute 3% of their gross monthly salary. The low salary, very high cost of living, and burden of other deductions from their salary were mentioned as the major reasons for the view. One participant stated that:

*“With my current income, contributing 3% is difficult. Nowadays, everything is expensive and I have a lot of other expenses such as house rent, food, school fee… for my family. I should not suffer to pay for SHI. I believe if you don’t wear clean cloths and eat right, you would get sick. It is unquestionable on the need to have SHI but the contribution should not lead us to further crisis and illness (Male, 60* *years, Teacher)”.*

Considering this burden, most of the respondents argued that 2% is enough contribution for the listed benefit packages. But a few of them were willing to pay more if the benefit packages would be revised. On the other hand, about one-fourth of the participants was willing to pay the 3% contribution and argued that a lesser premium would be an obstacle to achieving universal health coverage. However, community engagement and continuous discussions were suggested before implementation. One health professional stated:

*“If the contribution is too small, it is valueless as it can’t cover even the basic health services, let alone costly medications and diagnoses. Hence, the program will finally fail to succeed in its objective which could have unprecedented repercussions to everyone involved (Male, 40* *years, Teacher)”.*

Another participant stated that:

*“I am willing to contribute 3% but the problem is we don’t know the benefits and most of the time the government obliges public servants to contribute in many development plans without our consent. This is not a good approach. I think having a clear and genuine discussion is important to solve these ambiguities (Male, 40* *years, Teacher)”*.

A few of the health professionals claimed that they should not pay for services they provide, stressing that they are at high risk of infection or other harm and should therefore be entitled to get health services free of charge as compensation. One health professional argued that:

*“I shouldn’t contribute at all and it’s unfair if the government wanted us to accept it. I don’t. It is not fair to pay services that you can provide by yourself, as a health professional. Again, don’t forget that we are working in a risky environment. So, not only should I be treated freely but I should also be paid a hazard allowance for the possible risks while treating my patients (Male, 36* *years, Health professional)*”.

### Benefit packages

More than 1/3rd of participants argued that the list benefits included in the package don’t deserve a 3% contribution. Despite their agreement about excluding some services such as dentures and cosmetic surgery, most of them wanted chronic dialysis be included. A few participants agreed that out of country referrals for rare conditions should not be included as it contradicts the intention of health insurance to ensure access to basic health services for all citizens. One health professional supported this idea:

*“I do agree on the services excluded from the benefit package, because these disease conditions can consume a large amount of the budget for few patients that would have been used to save more lives. It is rational first to focus on conditions that affect a majority of the people (Male 33* *years, Health professional)*”.

### Quality of health services

Most participants rated the quality of health service in public health facilities as very poor and lower as compared to private health facilities. Almost all participants revealed their dissatisfaction with the current services which are characterized by a chronic shortage of medications and diagnostic supplies. Thus, all participants suggested current health services must be improved prior to implementation of the SHI. This was illustrated by one participant:

*“The current quality of health services is not optimal; there are challenges in receiving timely healthcare services. Again, we contribute to get services free of charge but from my experience medicines are frequently out of stock in public facilities and that means we will be forced to purchase them privately, as the private health facilities are not part of the scheme. It is frustrating if you paid and get nothing. It is better to improve the quality and availability of services prior to implementation, otherwise it might fail and it ultimately erodes public trust and will have unexpected repercussions (Male, 27* *years, Health professional)”.*

### Family members’ eligibility

Children above 18 years are not entitled to their parents’ health insurance benefits. The FGD participants were not happy with this age limit as it does not consider the economic situation of the child. Eligibility should not be based on age but individual income. In Ethiopia, most children of 18 years are the 11th or 12th grade, and some may go on to post-secondary education. Thus, coverage under their parents’ benefits should continue until approximately 23 years. One participant stressed that:

“*If the scheme excludes my family member above 18* *years, I don’t support this program. This doesn’t consider society’s real situation, I mean the chance of getting work is low and even at this age most of them are high school students. So, it should not only consider age but also it should consider level of income. Or if the government decided to exclude above 18* *years, there should be a means to create work for all citizens in that category (Male, 33* *years, Health professional)”.*

## Discussion

This study examined public servants’ preference of healthcare financing, WTP and factors affecting their WTP for the nationally proposed SHI scheme. A majority was in favor of a publicly funded program and was willing to pay an amount similar to that proposed by the government. Participants raised concerns about which health services would be funded, the quality and availability of health services, and age limits on coverage of dependents.

Understanding the preferences of civil servants’ healthcare financing options is important to estimate insurance uptake rate and implement a SHI scheme. Close to 90% of the respondents in the present study showed agreement on the need for SHI. This is relatively higher compared to previous studies in Ethiopia and elsewhere [20, 26–30]. The difference might be due to difference in study locations, time, awareness about the importance of social health insurance or due to increasing health care costs. This higher level of agreement on SHI has important health policy implication in that a majority of public servants would accept the envisaged healthcare financing option provided that some changes in the policy packages would be made.

The current survey revealed that the overall estimated mean WTP for SHI was 3.6% of respondents’ monthly salary, which is more than the premium currently proposed by the government [18]. It is, however, comparable to what was documented by a previous study conducted in Southern Ethiopia but higher than reports in Addis Ababa, the capital city [29, 31]. In contrast, most of our focus group discussants agreed to contribute about 2%. The differences among FGD and our survey could be attributed to the approaches used to illicit their WTP. During the FGD, participants were presented with a fixed 3% premium, the true contribution planned for the national SHI [18]. For the survey bid process, a number of different starting contributions were used and respondents were given hypothetical health insurance options. Evidence suggests people tend to demand low cost if they know the market values of that service [24].

This study found that age, educational status and household income were significantly associated with respondents’ WTP. Older age was associated with willingness to pay less, while respondents with higher household income were willing to pay more for SHI. This was in line with other studies conducted in Ethiopia, Uganda and Iran [20, 26, 32]. But findings were contrary to other studies which indicated that elderly people who had a higher risk of illness were willing to pay more [23, 27, 33]. This might be due to differences in economic status among elderly population across countries during retirement. Therefore finding ways to increase the income of public servants may positively increase their WTP.

It is interesting to note also that contrary to previous studies [20, 26, 27, 29, 32], in the present study more educated respondents showed willingness to pay less for SHI. This difference is due to the fact that our study population constituted health professionals who are largely getting healthcare free of charge, and hence contributing money, even if small amount, might be unacceptable to them.

Many other factors were indicated to affect WTP from the qualitative findings. The amount of premium and insurance benefit packages were among the most frequently raised issues during the discussion. Most participants revealed that about 2% is reasonable for the benefit package outlined in the current SHI policy but expressed willingness to contribute 3% provided the benefit packages would be revised to include some other services such as kidney dialysis. But few participants strongly opposed the inclusion of expensive services such as dialysis fearing that covering such services would drain the fund thereby compromising universal health coverage.

Quality of health services in public health facilities was an important issue in the focus groups. The majority of the participants were not satisfied with the availability and quality of health services in public facilities. They suggested that increasing the number of health professionals, improving medications and equipment supply, and bringing health services closer to the community would increase the acceptability of SHI. It is also important to note that implementation of SHI by itself could lead to increased patient load and further aggravate supply problems and affect the quality of health services [34–37]. This is an additional concern for policy makers to consider for the successful implementation of the SHI scheme.

Focus group discussants had concerns about ending benefits when a dependent turned 18 years old as per the recommendation of the current SHI policy [18]. All participants strongly contended that eligibility should not be based on age alone, but also on their capacity to generate income. Hence, further review of the plan and consultation with public servants is warranted before implementing SHI.

## Conclusion

The majority of participants in the studied area preferred SHI as their main approach to healthcare financing. Their mean WTP was 3.6% of their monthly salary, which is higher that the premium set by the government. This high acceptance rate and WTP has an important policy implication for the successful implementation of the scheme. Even though focus group discussants indicated a lower WTP, most discussants agreed to contribute more provided services are improved. Further dialogue with public servants is essential for successful uptake of the program.

## Additional files

**Additional file 1.** English version questionnaire.

**Additional file 2.** Focus group pre-discussion survey.

**Additional file 3.** Focus group discussion guide.

**Additional file 4.** Hypothetical health care financing scenarios.

## Abbreviations

DBDC: double bound dichotomous choice
FGD: focus group discussion
MOH: Ministry of Health
OOP: out of pocket
SHI: social health insurance
USAID: United States Agency for International Development
WHO: World Health Organization
WTP: willingness to pay
