# Supplementary material for: Willingness to pay for social health insurance and its determinants among public servants in Mekelle City, Northern Ethiopia: a mixed methods study
Source: Cost Eff Resour Alloc. 2019 Jan 15;17:2. doi: 10.1186/s12962-019-0171-x (PMC6332701; doi:10.1186/s12962-019-0171-x)
Supplement: Supplementary file 1 — Additional file 1. English version questionnaire. [file 12962_2019_171_MOESM1_ESM.docx]

**Additional file 1: English version questionnaire**

Willingness to pay for social health insurance and its determinants among civil servants in Mekelle city, Northern Ethiopia, participants’ interview questionnaire

Questionnaire No____________________________________________

Date of interview: day__________ month___________ year ___________

Interview times taken: started______________ ended________________

Working site: 1. Health facility 2. School

| **Part I: Socio demographic information** | | |
| --- | --- | --- |
| Q.N | Questions | Response Category |
| 1 | Gender | 1. Male  2. Female |
| 2 | Age (in years) | _____________ |
| 3 | Religion | 1. Orthodox  2. Muslim  3. Protestant  4. Catholic  5. Others (specify)__________ |
| 4 | Marital status | 1. Single  2. Married  3. Divorced  4. Widowed |
| 5 | Occupation | 1. teacher  2. health professional  3. supportive staff |
| 6 | Respondents net income per month | __________birr |
| 7 | Household income per month | __________birr |
| 8 | Household family size including you |  |
| 9 | Children 5 years and under | __________ |
| 10 | Children 6 to 18 years | __________ |
| 11 | Educational status | 1. no formal education  2. attend at least elementary school  3. Certificate  4. Diploma holder  5. Degree and above |
| **Part II:** Health Care Utilization Information | | |
| 12 | Do you or other member of the household have chronic illness (eg.DM, asthma, hypertension…)? | 1. Yes  2. No |
| 13 | Have any of your family members encountered illness during the last 12 Months? | 1. Yes  2. No |
| 14 | If yes when did the latest illness episode occur? | 1. Between 6 and 12 months ago  2. Between 3 and 6 months ago  3. Between 1 and 3months ago  4. Within 1 month |
| 15 | Have you encountered any illness during the last 12 months? | 1. Yes  2. No |
| 16 | If yes when did the latest illness episode occur? | 1. Between 6 and 12 months ago  2. Between 3 and 6 months ago  3. Between 1 and 3months ago  4. Within 1 month |
| 17 | How many times were you sick enough to seek care from healer or health professional in the past 12 months? |  |
| 18 | For how many days were you sick during the recent episode? |  |
| 19 | For how many days were you absent from your job due to the recent episode of illness? |  |
| 20 | Did you seek care for the recent episode? | 1. Yes  2. No |
| 21 | If yes for Q20 where did you get treated? | 1. Traditional healer  2. Local drug vender  3. Private Heath Facility  4. Public health facility  5. Others (specify) |
| 22 | Why did you go there? | 1. Considering the illness is self-limiting  2. easily accessible and convenient  3. Not expensive  4. Not too crowded  5. it is more effective  6. Didn’t have money to go elsewhere  7. Didn’t have time to go elsewhere  8. Others (specify)______ |
| 23 | How much did you pay in total for the visit? | ___________________Birr |
| 24 | How did you pay your medical care costs? | 1. Government (free)  2. Self (out of pocket)  3. family  4. private insurance  5. employer  6. Other_________________ |
| 25 | How did you see finding money to pay for the health care? | 1. Affordable  2. Un Affordable |
| 26 | If paying for a medical expense was difficult, how did you get it? | 1. Drawn from savings  2. Borrowing  3. Assisted by relatives  4. Undertaken extra work  5. Cut back on other things, food, school fees  6. Others (specify)_____________ |
| 27 | What is your satisfaction level with governmental health facility service quality? | 1. Very dissatisfied  2. Dissatisfied  3. Neutral  4. Satisfied  5. Very satisfied |
| 28 | What is your satisfaction level with governmental health facility service costs? | 1. Very dissatisfied  2. Dissatisfied  3. Neutral  4. Satisfied  5. Very satisfied |
| **Part III:Information related to participation in health insurance** | | |
| 29 | Have you heard about health insurance? | 1. Yes  2. No |
| 30 | If yes to Q29, what are the sources of information? | 1. mass media  2. Family  3. Friends  4. Insurance agency  5. Other (specify) ______ |
| 31 | Do you believe having health insurance is beneficial? | 1. Yes  2. No |
| 32 | If yes which of the following do you anticipate the benefits of health insurance? | 1. prevent from unexpected health expenditure  2. To help others who can’t afford their medical cost  3. to have timely care in times of illness  4. improve health services quality  5. Other(specify)______ |
| 33 | Do you have any kind of health insurance that covers your health expenditure? | 1. Yes  2. No |
| 34 | If yes for Q 33 which type of insurance coverage do you have? | 1.Private insurance  2.employement based insurance  3.others (specify)_____________ |
| 35 | If yes to Q33, how many members of your household (including you) covered by a health insurance? |  |
| 36 | If yes to Q33, what is the amount of money you pay for health insurance per month for the whole family? |  |

**Part III: health system financing scenario**

Now I will present three financing systems for health care. Please identify which health financing you are willing to join.

**Scenario A:No insurance (out-of-pocket model):** you will pay the full cost for each visit to health institution and for the medicine prescribed to you and your families. If you are not able to pay you will not receive any service. A service is given at cost price. There are not either any exemption cards (free service). The total annual cost for a household depends on how many family members that will be ill and visit the health institution.

**Scenario B:Compulsory health insurance:** All employees are compulsorily obliged to pay and contributed a monthly premium (fee) to a health care fund. There are not either exemption cards. The fee is based on deducting specified percentage of their monthly salary determined by insurance agency. Thereby the employees spouse and family members of less than 18 years are entitled to free health care at a nearby health center and free medicine if prescribed by a doctor. The fund will be managed through an independent health care fund. If care at a higher level is needed, the insured patient will be supported and entitled to free health service in these facilities.

**Scenario C.voluntary health insurance:**Each employee/household can choose to voluntarily pay a monthly premium (fee) to an insurance organization. For a household the fee is based on the number of beneficiary in the household and number of health facility visit. All persons in the household paying the fee are entitled to free health care at a nearby healthfacility and free medicine if prescribed by a doctor. If care at a higher level isneeded, the insured patient will be supported by an amount based on the cost perday at the nearby health center.

| 37 | Which systems do you prefer? | 1. A  2. B  3. C |
| --- | --- | --- |
| 38 | Do you think the government should set up the type of health insurance stated in B? | 1. Yes  2. No |
| 39 | If most people prefer B and the system would be implemented, are you willing to join the insurance? | 1. Yes  2. No |
| 40 | If yes to Q39, what is your reason for your answer? | 1. It provides free access to medical care at point of service  2. I need to help others who can’t afford their medical costs  3. For security and peace of mind in times of ill-health  4. I am facing health problem frequently  5. Other(specify)______ |
| 41 | If your answer is no to Q39, what is your reason for your answer? | 1. I do not have enough money to pay  2. insurance package doesn’t cover all health services  3. OOP charge is better  4. Lack of trust in government programs  5. Lack of trust in insurance scheme  6. poor quality of health service in government health facility  7. Contributing money for sickness in advance is a taboo  8. others (specify) |
| 42 | If the insurance premium will be [6%, 4%, 3% or 2%] of your monthly income (deducted from your gross salary) are you willing to join the insurance? | 1. Yes  2. No |
| 43 | If your answer is yes for Q42 would you pay double of the above specified amount? | 1. Yes  2. No |
| 44 | If answer is no for Q42 will you pay half of the amount specified in Q42? |  |
| 45 | If your answer is no for Q44 what is your reason? | 1. I do not have enough money to pay  2. insurance package doesn’t cover all health services  3. it excludes family member after 18  4. I doubt the management of the fund  5. Other rich members of the society should pay more  6. others (specify) |
| 46 | Maximum how much percent of your gross monthly salary are you willing to pay? | __________% of your monthly salary |
| 47 | If your answer is 0% for Q46 what is your reason? | 1. I doubt the management of the fund  2. It is the responsibility of the government to pay for such a programme  3. Because of lack of money  4. Other rich members of the society should pay for the programme  5. Other (specify)________________ |

**48. Do you have comments regarding social health insurance?**
