# Supplementary material for: Willingness to pay for social health insurance and its determinants among public servants in Mekelle City, Northern Ethiopia: a mixed methods study
Source: Cost Eff Resour Alloc. 2019 Jan 15;17:2. doi: 10.1186/s12962-019-0171-x (PMC6332701; doi:10.1186/s12962-019-0171-x)
Supplement: Supplementary file 2 — Additional file 2. Focus group pre-discussion survey. [file 12962_2019_171_MOESM2_ESM.docx]

**Additional file 2: Focus Group Pre-Discussion Survey**

Focus group site____________________________________________

Participant’s numerical identifier __________________________

Date of focus group session ______________________________

| Q.N | Questions | Response category |
| --- | --- | --- |
| 1 | Gender | 1. Male  2. Female |
| 2 | Age (in years) | **_________**year |
| 3 | Marital status | 1. Single  2. Married  3. Divorced  4. Widowed |
| 4 | Occupation | 1. teacher  2. health professional  3. lower paid supporting staff |
| 5 | Educational status | 1. no formal education  2. attend at least elementary school  3. Certificate  4. Diploma holder  5. Degree and above |
| 6 | Your average income per month | ____________ETB |
| 7 | Your average household income per month | ____________ETB |
| 8 | Household family size |  |
| 9 | Children under 5 years |  |
| 10 | Children 6 to 18 years |  |
| 11 | Which health facility do you utilize most of the time? | 1.private  2.public |
| 12 | Do you have any kind of health insurance or organizations that covers your health expenditure? | 1. Yes  2. No |
| 13 | If your response to Q #10 is yes, which type of insurance coverage do you have? | 1.Private health insurance  2.employement based health insurance  3.others (specify)_____________ |
| 14 | How much did you spent at household level on health care service over the past year? | _________________ETB |
| 15 | What is the maximum amount that you would be willing to pay for SHI (deducted from your gross salary) for you and your family members, that included the benefits discussed during today’s focus group session? | _________% of my monthly salary. ( **Please answer this question at the end of our discussion**) |
