# Supplementary material for: Willingness to pay for social health insurance and its determinants among public servants in Mekelle City, Northern Ethiopia: a mixed methods study
Source: Cost Eff Resour Alloc. 2019 Jan 15;17:2. doi: 10.1186/s12962-019-0171-x (PMC6332701; doi:10.1186/s12962-019-0171-x)
Supplement: Supplementary file 3 — Additional file 3. Focus group discussion guide. [file 12962_2019_171_MOESM3_ESM.docx]

**Additional file 3: Focus group discussion guide**

Focus group site______________________________________________________

Facilitator ____________________________ Recorder_____________________

Start time___________________ End time ________________ Duration _______

FGD Guide

1. How do you pay yours or your families’ healthcare expenditures? Is there an organization that covers part or full of your health care expense?

Probe: if it is out of pocket, how do you comment on the affordability of services?

Probe: if it is partially/fully covered by another organization, how do they make the payment (mode of payment and coverage)?

Probe: What portions of your family member are covered?

Probe: Does the organization cover every service fee? If not, what services are excluded from or include in the benefit package? What do you think is the reason for the exclusion?

Probe: are you satisfied with the type of health insurance that you have currently? If not, why? Would you prefer to have other options? Explain

1. What comes to your mind when someone says health insurance?What does health insurance mean to you? How do you think it works?

Probe: what kind of health insurance are you familiar with?

Probe: what do you think about the purpose of health insurance?How do you describe the benefits of health insurance? Who do you think benefited more and why?

Now let me give you some highlight about Ethiopian health insurance scheme and its benefit packages.

Due to increased cost of health care services, it becomes difficult to provide quality health care for the larger community. In addition, there are poor population segments that are not able to pay out of pocket health care costs.The purpose of health insurance is, therefore, to share the risk among the relatively rich with those poor and the healthy with those sick. It is an important tool for insuring universal health coverage and in improving health service. Health insurance protects families financially in the event of an unexpected serious illness or injury that could be very expensive.

Health insurance provides individuals and families access to health facility services with little or no out-of-pocket expenditures at the point of service. This is accomplished by requiring that individuals or families pay a small portion of their income, usually per month, into a health insurance fund. By doing so, the individuals and/or his family members are guaranteed financial access to health care services when needed.” There are two health insurance plan proposed by government, currently. Community health insurance to cover citizens engaged in informal sectors; mainly those dwellers of rural areas and social health insurance for formal sector workers including you and pensioners. The following are benefit packages and services excluded from the insurance scheme

| **The benefit packages** | **Services excluded** |
| --- | --- |
| Outpatient care | Any treatment outside Ethiopia |
| inpatient care | Health services provided free of charge to any beneficiary like MCH, ART, TB |
| delivery services | Treatment related to drug abuse or addiction |
| surgical service | Periodic medical checkup unrelated to illness |
| diagnostic tests and generic drugs included in the drug list of the agency and prescribed by practitioner | Cosmetic surgery  Organ transplants |
|  | Dialysis except acute renal failure |
|  | Provision of eye glass and contact lenses, hearing aids |
|  | Dentures, implants, crowns |
|  | Epidemics |

1. Now, that you understand the purpose of health insurance.Do you think Ethiopia needs social health insurance? Why or why not?
2. Would you be willing to join and pay for the social health insurance at your current income level and pay 3% of your gross monthly income required to keep you eligible for health benefits?

**Probe:**If yes, what makes you decide to join and contribute for SHI?Do you think the 3% contribution is fair or not? Why?(Cost, health service quality, health status, risk sharing (helping others who can’t afford their medical bills, financial security in times of ill health…)

**Probe**: If you are not willing to join and contribute for SHI, what is your reason? (Can’t afford, government should pay, service coverage, service quality, lack of trust….)

**Probe:** If your income level were to increase by some amount, would you be willing join for SHI and pay the monthly premium required? Why?

**Probe:** If you think the 3% contribution is expensive, what percent of your gross salary would you be willing to contribute?

**Probe:**what do you think as a fair system of financing for health care?

1. What do you think the government should do that might be comfortable for purchasing social health insurance?

Probe: (reduce the premium; revise the benefit package, improve service quality, include private health institution….)

1. Does anyone have any other comments on today’s discussion?
