# Supplementary material for: Willingness to pay for social health insurance and its determinants among public servants in Mekelle City, Northern Ethiopia: a mixed methods study
Source: Cost Eff Resour Alloc. 2019 Jan 15;17:2. doi: 10.1186/s12962-019-0171-x (PMC6332701; doi:10.1186/s12962-019-0171-x)
Supplement: Supplementary file 4 — Additional file 4. Hypothetical health care financing scenarios. [file 12962_2019_171_MOESM4_ESM.docx]

**Additional file 4: hypothetical health care financing scenarios**

| **Scenario A**.No insurance (out-of-pocket model):Employee pays the full cost for each visit to health institution and for the medicine if the patient is prescribed medicine. If not able to pay will not receive any service. A service is given at cost price. There are not either any exemption cards (free service). The total annual cost for a household depends on how many family members that will be ill and visit the health institution. |
| --- |
| **Scenario B**.Compulsory health insurance: All employees are compulsorily obliged to pay and contributed a monthly premium (fee) to a health care fund. There are not either exemption cards. The fee is based on deducting specified percentage of their gross monthly salary determined by insurance agency. Thereby the employees spouse and family members of less than 18 years are entitled to free health care at a nearby health center and free medicine if prescribed by a doctor. The fund will be managed through an independent health care fund. If care at a higher level is needed, the insured patient will be supported and entitled to free health service in these facilities. |
| **Scenario C.**voluntary health insurance:Each employee/household can choose to voluntarily pay a monthly premium (fee) to an insurance organization. For a household the fee is based on the number of beneficiary in the household and number of health facility visit. All persons in the household paying the fee are entitled to free health care at a nearby healthfacility and free medicine if prescribed by a doctor. If care at a higher level isneeded, the insured patient will be supported by an amount based on the cost perday at the nearby health center. |
